# Electrocatalytic and Enhanced Photocatalytic Applications of Sodium Niobate Nanoparticles Developed by Citrate Precursor Route

**DOI:** 10.1038/s41598-019-40745-w

**Published:** 2019-03-14

**Authors:** Umar Farooq, Ruby Phul, Saad M. Alshehri, Jahangeer Ahmed, Tokeer Ahmad

**Affiliations:** 1 Nanochemistry Laboratory, Department of Chemistry, Jamia Millia Islamia, New Delhi, 110025 India; 2 Department of Chemistry, College of Science, King Saud University, Riyadh, 11451 Saudi Arabia

## Abstract

Development of cost effective and efficient electrocatalysts is crucial to generate H_2_ as an alternative source of energy. However, expensive noble metal based electrocatalysts show best electrocatalytic performances which acts as main bottle-neck for commercial application. Therefore, non-precious electrocatalysts have become important for hydrogen and oxygen evolution reactions. Herein, we report the synthesis of high surface area (35 m^2^/g) sodium niobate nanoparticles by citrate precursor method. These nanoparticles were characterized by different techniques like X-ray diffraction, transmission electron microscopy and X-ray photoelectron spectroscopy. Electrocatalytic properties of cost-effective sodium niobate nanoparticles were investigated for HER and OER in 0.5 M KOH electrolyte using Ag/AgCl as reference electrode. The sodium niobate electrode showed significant current density for both OER (≈2.7 mA/cm^2^) and HER (≈0.7 mA/cm^2^) with onset potential of 0.9 V for OER and 0.6 V for HER. As-prepared sodium niobate nanoparticles show enhanced photocatalytic property (86% removal) towards the degradation of rose Bengal dye. Dielectric behaviour at different sintering temperatures was explained by Koop’s theory and Maxwell-Wagner mechanism. The dielectric constants of 41 and 38.5 and the dielectric losses of 0.04 and 0.025 were observed for the samples sintered at 500 °C and 700 °C, respectively at 500 kHz. Conductivity of the samples was understood by using power law fit.

## Introduction

With every passing day, demand for global energy is growing exponentially, which has sparked intense research to develop sustainable, efficient energy resources and better storage mechanisms. Recently researchers have focussed on to develop materials having diverse applications. So as a consequence, there is great surge in development of active materials for energy generation purposes and storage applications. In view of the energy generation, hydrogen is considered as an alternative energy source for next generations which could replace conventional fossil energy sources^1,2^. While as for energy storage and energy transfer processes, materials with high and stable value of dielectric constant and low dielectric loss are gaining interest^3^. For energy generation, splitting of water through the processes like photocatalysis and electrocatalysis is considered as promising, non-toxic and environment friendly way for production of hydrogen^4–7^. During electrocatalytic water splitting, hydrogen (H_2_) is produced through hydrogen evolution half-cell reaction (HER) and oxygen (O_2_) is evolved via oxygen evolution half-cell reaction (OER). However, the state-of-art platinum-based materials for HER and noble metal electrocatalysts like IrO_2_ and RuO_2_ for OER acts as bottleneck for large scale commercial application because of their scarcity and precious nature. Another important reason that hinders the practical application of electrocatalysis is that the OER needs large over potential due to complex four step proton coupled electron reaction mechanism. Even after the use of active catalysts like IrO_2_ and RuO_2_, OER shows sluggish kinetics^8–10^. In comparison to OER, HER is only two electron transfer process hence requires low energy (over potential) to overcome the kinetic barrier. To date, IrO_2_ and RuO_2_ are considered as most appropriate electro OER catalysts due to their low over potential, stability and excellent activity^11–13^. Consequently, the design of cheap, highly efficient and earth abundant materials for HER and OER is the main aim of the current research in the field of renewable energies^14,15^. Another major shortcoming of most of the electrocatalysts is that either they are active in acidic medium for HER or in alkaline medium for OER reaction. Therefore, it is imperative to develop a catalyst which could show both HER and OER simultaneously either in acidic or alkaline medium only.

Extensive research has been carried out to understand the electrocatalytic processes and to develop cheap, stable and efficient alternate bifunctional electrocatalytic materials for hydrogen evolution (HER) and oxygen evolution reactions (OER). With the understanding of electrocatalytic processes, transition metal oxides, sulphides and phosphides have been vastly explored as an important effective bifunctional material for electrocatalysis^16–21^. Perovskites having general formula ABO_3_ are well known cost-effective materials with huge composition and electronic diversity. In ABO_3_ type perovskites, rare earth or alkaline earth metals occupy site “A” while as site “B” is occupied by any transition metal. These materials are of great significance due to their comparable OER activities with respect to gold standards of electrocatalysis like IrO_2_ and RuO_2_. Perovskites have shown fast growth as oxygen transporting membranes and are considered as promising electrocatalytic materials for fuel cells^22–25^. By employing filled orbital state of e_g_ as an OER electrocatalytic activity descriptor for transition metal ions, perovskite Ba_0.5_Sr_0.5_Co_0.8_Fe_0.2_O_3_ (BSCF) shows an optimum e_g_ orbital occupancy equal to unity with a maximum intrinsic OER activity with respect to IrO_2_ and RuO_2_. However, the main issue which hinders the commercial application of BSCF is leaching i.e. it readily becomes amorphous by producing Co-O motifs under OER condition^24–26^. Recently, May *et al*.^26^, had reported new family of double perovskites Ln_0.5_Ba_0.5_CoO_3_ (Ln = Pr, Nd, Sm, Gd) with stacking layers which shows enhanced electrocatalytic properties due to their intrinsic activity, the highest activity was found in Pr_0.5_Ba_0.5_CoO_3_ (PBC). However, the electrocatalytic OER activity was reduced by 7% when tested for 25 cycles of cyclic voltammetry. Similarly, for HER large number of alternative materials like transition metal phosphides, selenides, sulphides, nanocomposites have been explored as possible substitutes for noble metal electrocatalysts. For electrocatalytic processes, small nanosized, porous and hollow materials are considered highly favourable, as they provide high surface area to volume ratio, kinetically desirable structures with short ion diffusion length^27,28^.

In addition to green energy generation, environment remediation is another important concern for researchers. Annually 0.7 million tons of toxic dyes having organic nature are generated globally and discharged into the environment^29^. For textile, printing and photochemical applications, xanthene dyes such as Rose Bengal are generally employed. It is highly toxic dye which not only causes irritation of skin, respiratory tract but also is highly toxic to corneal epithelium^30^. So, it is of prime importance to efficiently remove this toxic dye from industrial wastewater effluents. So far, many chemical, biological and physical approaches have been adopted to eliminate harmful organic dyes from waste water. Among these, the most promising approach to remove the organic dyes includes adsorption and photocatalytic technology^31,32^. Photocatalysis is an important process that has acclaimed global interest in environmental remediation and water splitting processes. In recent years, nanostructured semiconductors have gained lot of interest as photocatalysts for remediation of waste water from industrial effluents and organic pollutants like dyes. Followed by the landmark discovery of photocatalytic application of TiO_2_ by Fujishima and Honda, the semiconductor photocatalysts have acclaimed wide global interest for researchers^33^. Now a days, efforts are now being made to surpass the photocatalytic efficiency of TiO_2_ semiconductor photocatalyst. Therefore, new photocatalyst materials such as ZrO_2_, ZnO, NaNbO_3_, SnO_2_, CuCrO_2_, YFeO_3_ and so on have been explored^34–40^. Among these different nanostructure photocatalysts, ternary oxides having perovskite structure like alkali niobates and tantalates (NaNbO_3_, NaTaO_3_), SrTiO_3_ have evolved interesting environmental effective alternate materials to TiO_2_ for elimination of environmental poisonous pollutants^41–43^.

Sodium niobate is an important n-type perovskite material possessing wide range of applications. Due to structural flexibility, NaNbO_3_ shows polymorphism over a wide range of temperature and possess important physical, chemical characteristics due to which it has been applied in vast advanced technologies like photocatalysis, piezoelectric sensors and electronic devices. The properties and applications of NaNbO_3_ nanoparticles are highly affected by its morphology, size and structure. The important method to boost the properties of these materials is to improve the surface area by reducing the size. A variety of methods like reverse micelle, sol-gel, solvothermal, hydrothermal and polymeric citrate precursor methods etc. have been explored to synthesize simple and complex nano-sized materials^44–47^. Herein, we report the low temperature synthesis of high surface area perovskite sodium niobate (NaNbO_3_) nanoparticles by polymeric citrate precursor (PCP) method. The main advantage of using PCP route is that bulk amount of cost effective, less toxic and highly crystalline nanoparticles with enhanced properties could be synthesized at very low temperature compared to solid state route. In PCP method, no inert conditions and sophisticated instruments are required during the reaction process^37,40^. Sodium niobate has been previously investigated for its photocatalytic, piezoelectric properties. However, in this paper we have designed sodium niobate nanoparticles with high surface area by citrate precursor route. The main aim of the work was to develop an efficient catalyst having diverse usage for energy generation and storage purpose. Here, we explored both photocatalytic as well as electrocatalytic properties of these nanoparticles. To the best of our knowledge, the electrocatalytic application of NaNbO_3_ is first time studied in this report which shows the promising results for both OER and HER in alkaline medium. NaNbO_3_ shows transition from antiferroelectric phase with orthorhombic structure at 25 °C to ferroelectric phase at 280 °C^48^. To fully understand the different structural changes of NaNbO_3_, different studies like dielectric, pyroelectric have been carried out^49^. There are many reports on dielectric studies of NaNbO_3_^49,50^ but, the detailed investigation of variation of dielectric properties and conductivity with frequency and temperature has not been done so far. The detailed investigation of dielectric properties was also done which shows that NaNbO_3_ may act as promising high temperature dielectric material due to its stable dielectric properties.

## Results and Discussion

### Structural characteristics

**Figure 1:**
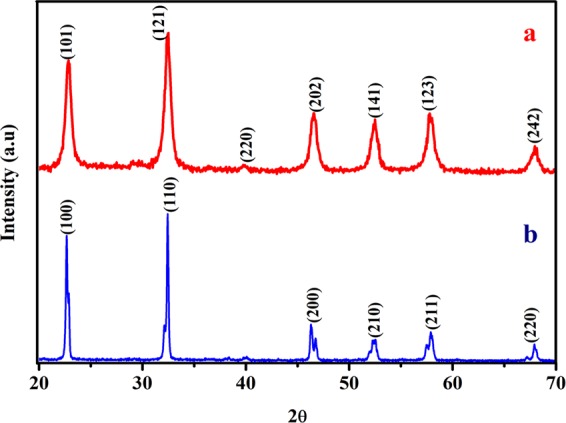
XRD pattern of (**a**) NaNbO_3_ nanoparticles prepared by PCP route and (**b**) bulk NaNbO_3_.

The phase composition, crystallinity and purity of the samples was analysed by X-Ray diffraction technique. The XRD result of the precursor obtained at 300 °C is given in Fig. S1. All the observed diffraction peaks of the precursor correspond to the standard JCPDS card No. 731788 having chemical composition of NaNb_13_O_33_. The XRD results of NaNbO_3_ nanoparticles obtained at 500 °C is shown in Fig. 1a. From XRD profile, seven diffraction peaks appear at 22.88, 32.42, 39.79, 46.57, 52.38, 57.79 and 68.01 degrees, were indexed to (101), (121), (220), (202), (141), (123) and (242) lattice planes, respectively. It was revealed that the diffraction peaks observed in XRD correspond to the NaNbO_3_ having orthorhombic phase, with lattice parameters (a = 5.569 Å, b = 7.790 Å, c = 5.518 Å) and space group P2_1_ma (JCPDS No. 742454). The XRD pattern shows that the synthesized NaNbO_3_ nanoparticles are highly crystalline and no phase other than orthorhombic NaNbO_3_ have been formed. Bulk NaNbO_3_ was also synthesized to compare the electrocatalytic properties. The XRD of bulk NaNbO_3_ is shown in Fig. 1b, which matches with NaNbO_3_ (JCPDS No. 895173) possessing orthorhombic phase structure. The intensity of reflections appeared for bulk NaNbO_3_ is found to be high as compared to NaNbO_3_ nanoparticles synthesized by polymeric citrate precursor route, which is due to high crystallinity and large particle size of the sample.

### Electron microscopic studies

**Figure 2:**
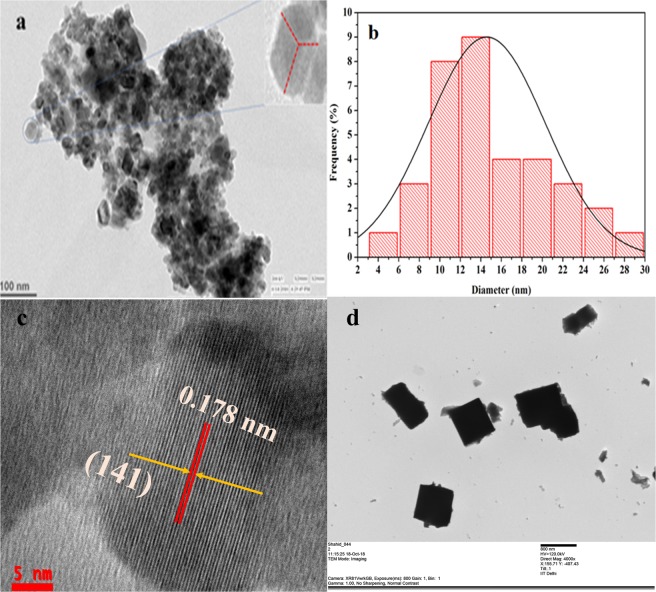
(**a**) TEM micrograph, (**b**) size distribution histogram and (**c**) HRTEM of NaNbO_3_ nanoparticles synthesized by PCP and (**d**) TEM image of bulk NaNbO_3_ particles.

The electron microscopic study was carried out by using TEM technique. A uniform dispersion of synthesized samples in water for TEM analysis was prepared by ultrasonication for 20 min and sample preparation was done by drop casting of a dispersed sample on copper grid. Figure 2a represents the TEM micrograph of as-synthesized NaNbO_3_. Figure 2b represents the size distribution histogram of the as-synthesized nanoparticles. TEM micrograph shows that nanoparticles having size in the range of 5–30 nm were synthesized and average size of particles was found to be 15 nm. Therefore, the TEM result shows that synthesis of NaNbO_3_ could be effectively controlled to nanoscale level using polymeric precursor method. TEM image also shows that the non-uniformly synthesized nanoparticles have different geometries but, in TEM image orthorhombic shaped nanoparticles with a ≠ b ≠ c is also apparently visualized in the inset of Fig. 2a. The lattice fringes visible in HRTEM image could be ascribed to NaNbO_3_ and the d spacing was found to be 0.178 nm which corresponds to the (141) crystal plane of NaNbO_3_ nanoparticles as shown in Fig. 2c. TEM micrograph of bulk NaNbO_3_ particles as shown in Fig. 2d suggests large particle size as compared to the nanoparticles synthesized by PCP route.

### X-ray photoelectron spectroscopic (XPS) analysis

**Figure 3:**
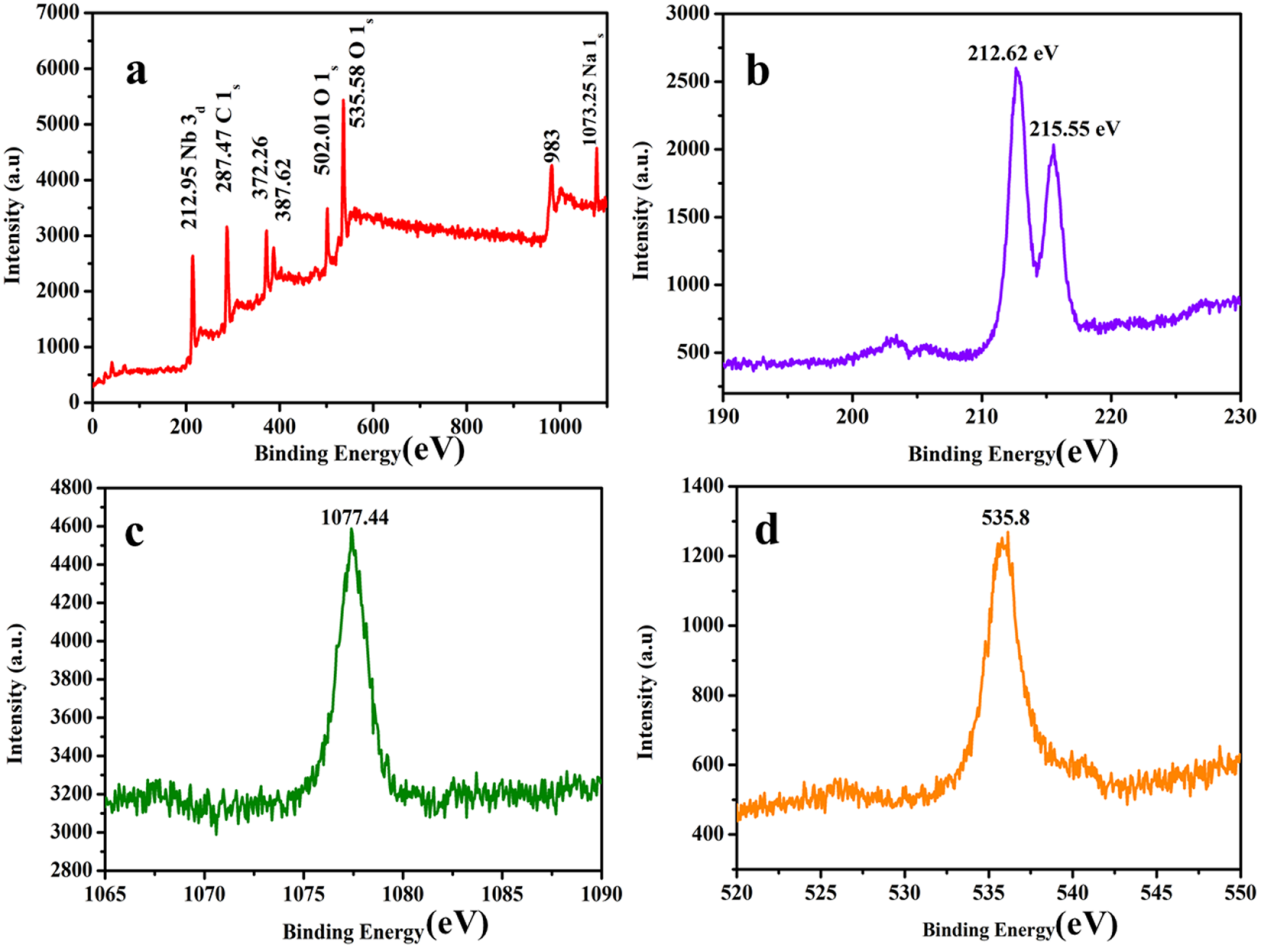
Full range XPS survey spectrum of (**a**) NaNbO_3_ photocatalyst, (**b**) high resolution Nb3d spectrum, (**c**) high resolution Na1s spectrum and (**d**) high resolution O1s spectrum.

XPS was employed to evaluate the chemical state and chemical composition of as-prepared NaNbO_3_ photocatalyst. Figure 3a represents the wide scan XPS spectrum of NaNbO_3_ nanoparticles. From XPS, it was observed that synthesized nanoparticles consist of Nb, Na, O and C. Full range XPS spectra show peaks for Na-1*s*, O-1*s*, Nb-3*d* and C-1*s*. Figure 3b displays the high resolution specific XPS spectra of Nb 3d and shows two signals positioned at 212.6 and 215.5 eV. These peaks correspond to 3d doublet of Nb at 212.6 for Nb 3d_5/2_ and at 215.5 eV for Nb 3d_3/2_. From peaks it is clear that there is no satellite peak present with Nb which is present in +5 chemical state in the synthesized material. The high resolution XPS spectra for Na as shown in Fig. 3c, shows the peak at 1077.4 eV which demonstrates that Na 1*s* exists as single peak which indicates that Na ions have +1 chemical state in the prepared nanoparticles. Similarly, the high-resolution peak for O-1*s* is shown in Fig. 3d. Only one single peak is observed in high resolved XPS spectra of oxygen showing that O is present in single atmosphere in as-prepared compound. The peak observed at 535.8 eV showing that O is attached to Nb and Na ions in NaNbO_3_.

### BET surface area analysis

**Figure 4:**
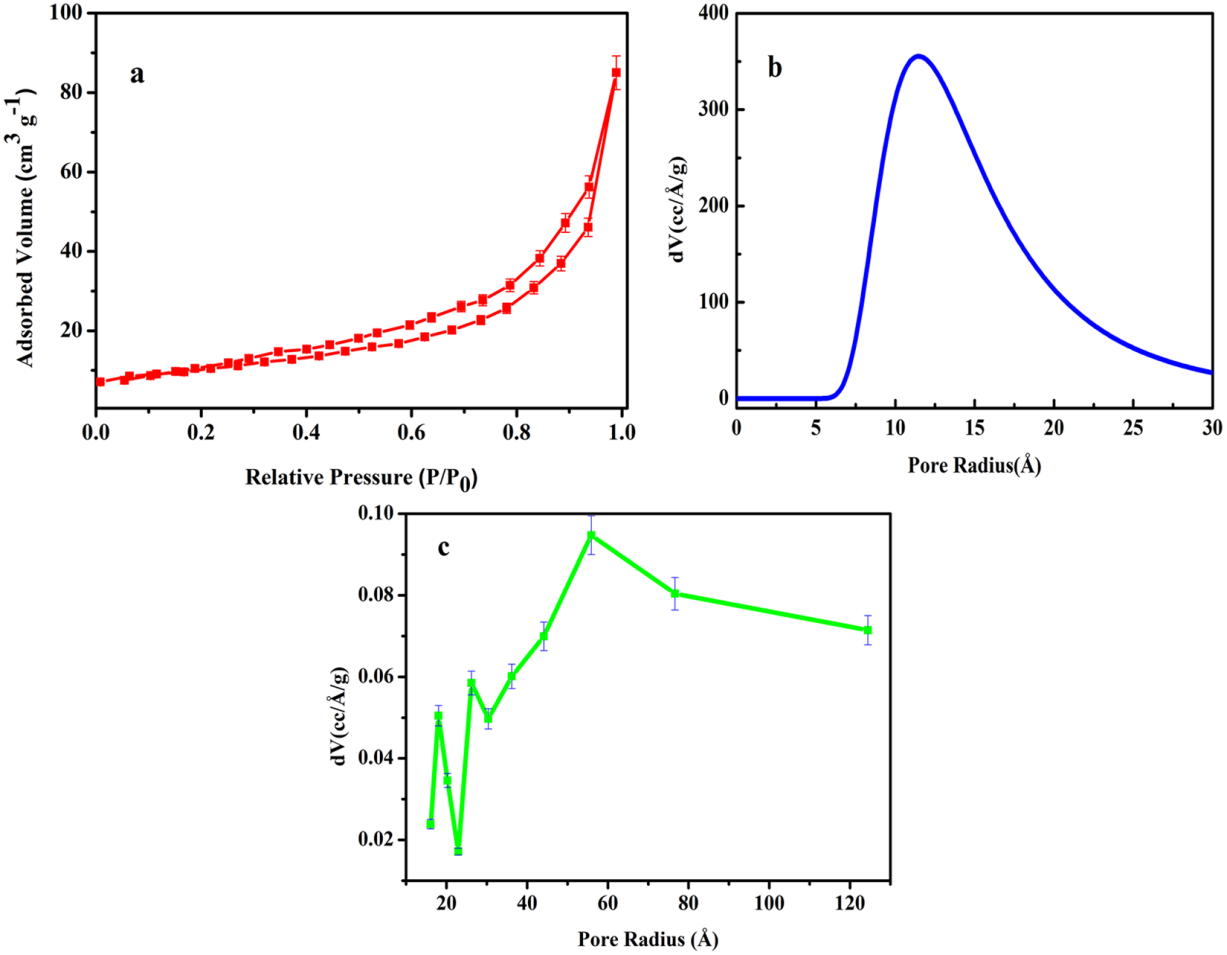
(**a**) Nitrogen adsorption/desorption isotherm, (**b**) DA average pore size distribution plot and (**c**) BJH pore size distribution plot of NaNbO_3_ nanoparticles.

Surface area is an important factor which influences the electrocatalytic and photocatalytic activities of the catalysts. Therefore, before discussing the electrocatalytic and photocatalytic properties of the synthesized sample, analysis of surface area is of primary importance. In general, larger the surface area of the catalyst greater is its catalytic activity. This is because, materials with high surface area offer more surface-active sites which leads to increase in adsorption and reaction sites for catalytic process, leading to an improvement in photocatalytic and electrocatalytic processes. Large surface area besides increasing the active sites for adsorption but reduces the length for ionic diffusions thus, provides the kinetically suitable structures for electrocatalysis. To study the surface area and pore size of the sample, N_2_ adsorption-desorption measurements were done using multipoint BET method. Figure 4a represents the N_2_ adsorption-desorption curves of NaNbO_3_ nanoparticles, which follows the type III isotherm indicating that additional adsorption is taking place due to strong interaction between the adsorbent and the adsorbed layer as compared to interaction between adsorbate and the adsorbent surface i.e. multilayer adsorption is taking place in the adsorbed molecules clustered at more favourable sites^51^. Hysteresis of such type is generally formed by the solids consisting of aggregated or agglomerated particles which lead to the formation of slit shaped pores having nonuniform size or shape. The specific surface area for NaNbO_3_ nanoparticles was found to be 35 m^2^/g, which is higher than the data present in the literature^52–54^. High surface area is attributed to the reduction in size to nanoscale range. Dubinin-Astakhov (DA) pore size distribution of NaNbO_3_ was deduced from the plot using both adsorption and desorption data points as shown in Fig. 4b. The distribution of pore size was found to be 7.5–22.5 Å and the onset of the plot was found to be at 11.5 Å, which confirms the mesoporous characteristic of the sample. From the adsorption points of the sample, the average pore size distribution was determined by Barrett-Joyner-Halenda (BJH) plot as shown in Fig. 4c. Derived from the calculations using BJH, the sample shows wide average pore size distribution centred at ≈58 Å. The results obtained show that mesoporosity is enhanced using citrate precursor route, particularly due to the pre-occupied space by precursor. Although pre-occupied space by precursor is not directly involved in the pore formation therefore it affects the porosity of the nanoparticles as reported by Chen *et al*.^55^ which is attributed to the increased surface area of the material.

### OER catalytic performance of sodium niobate

**Figure 5:**
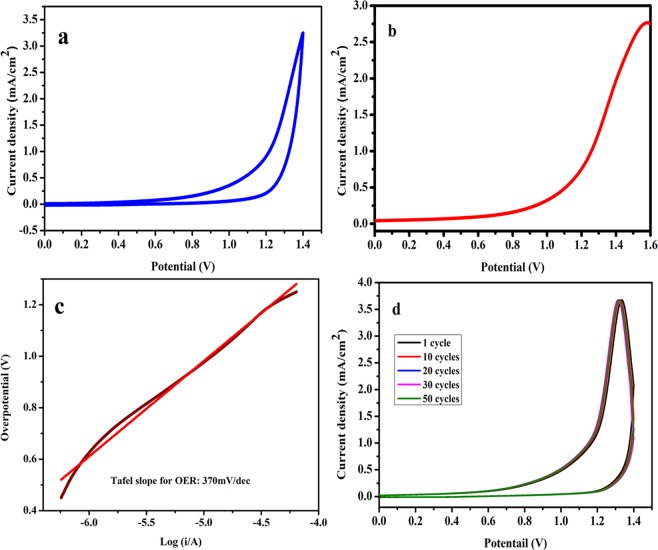
(**a**) Cyclic voltammograms (CV). (**b**) Linear sweep voltammetry (LSV) curve in O_2_ saturated 0.1 M KOH solution at a scan rate of 100 mVs^−1^. (**c**) Tafel plot and (**d**) CV plot for stability determination of NaNbO_3_ nanoparticles for OER activity.

Our primary interest in alkali niobates is to study their electrocatalytic behaviour for OER at room temperature. The nanosized NaNbO_3_ should exhibit certain degree of catalytic property due to its high surface area and porous nature as confirmed from BET studies, which could allow the ionic species to diffuse easily and hence promotes effective consumption of active sites. Figure 5a shows the cyclic voltammetry plot of NaNbO_3_ nanoparticles. Nanosized NaNbO_3_ start generating current at 1.09 V vs Ag/AgCl at a scan rate of 100 mV/s and above this potential it acts as electrocatalyst for OER. The amount of oxygen evolved during the electrocatalytic process is directly proportional to the intensity of the anodic peak current.

Linear sweep measurements for OER were carried out at a scan rate of 100 mV/s by sweeping the potential across negative to positive value ranging from −0.2 to 1.8 V. Figure 5b represents a typical polarization plot of the sodium niobate electrocatalyst, which exhibits significant OER activity with onset potential of 0.9 V with respect to Ag/AgCl electrode and above this potential there occurs an abrupt rise in the anodic current as a result of O_2_ evolution. The obtained onset potential is comparable to the art-of-state electrocatalysts which include RuO_2_ (≈1.45 V), IrO_2_ (≈1.50 V) and double perovskite-based catalyst like Ba_0.5_Sr_0.5_Co_0.8_Fe_0.2_O_3_ (BSCF ≈1.50 V), Pr_0.5_Ba_0.5_CoO_3_ (≈1.50 V)^26,56–63^. Nanosized NaNbO_3_ show significant electrocatalytic activity (2.7 mA/cm^2^ in alkaline medium) compared to IrO_2_ and RuO_2_ which shows current density of (~4 µA/cm^2^ in acidic and ~2 µA/cm^2^ in basic electrolyte solution) and (~10 μA/cm^2^ in acidic and ~3 μA/cm^2^ in alkaline electrolyte solution) respectively^58^. To compare the activity of nanosized high surface area NaNbO_3_, the electrocatalytic activity of bulk NaNbO_3_ with large particle size was also studied. Figure S2a represents the LSV plot of bulk NaNbO_3_. LSV plot shows very small current was generated (0.000004 mA/cm^2^) from bulk NaNbO_3_ surface, which confirms that NaNbO_3_ particles having small size and high surface area show better electrocatalytic activity compared to bulk NaNbO_3_. More insight into the OER activity was gained by studying the kinetic performance of sodium niobate for OER activity by employing the tafel plot, which is obtained from the polarization curve using the tafel equation

$${\rm{\eta }}={\rm{b}}\times \,\mathrm{log}\,(J/{J}_{o})$$

where η, b, *J* and *J*_*o*_ corresponds to the over potential, tafel slope, current density and exchange current density of the electrocatalytic reaction respectively. The tafel plot of sodium niobate nanoparticles and bulk NaNbO_3_ is shown in Figs 5c and S2b. The tafel slope value for nanosized and bulk NaNbO_3_ was equal to 370 and 409.6 mV/decade respectively. The resulting tafel slope value of NaNbO_3_ synthesized by PCP route is comparable to Pt based electrocatalyst^61^, but larger than the standard electrocatalysts like RuO_2_ and IrO_2_^62,63^, whereas for bulk NaNbO_3_ tafel slope is too high showing slow reaction rate for OER. For long term functioning of the electrode, the stability of the material is much more crucial. Herein, the stability of NaNbO_3_ electrode material was checked by Cyclic voltammetry at 100 mV/s upto 50 cycles at a potential window ranging from 0 to 1.65 V. Figure 5d shows that the catalyst displays little loss of activity, confirming the stability of the electrode material.

**Figure 6:**
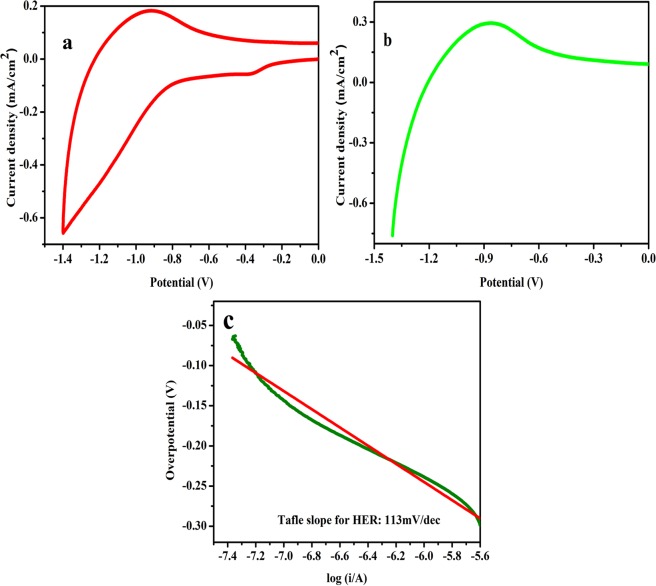
(**a**) Cyclic voltammograms (CV). (**b**) Linear sweep voltammetry (LSV) curve in O_2_ saturated 0.1 M KOH solution at a scan rate of 100 mVs^−1^. (**c**) Tafel plot of NaNbO_3_ nanoparticles for HER activity.

The catalytic activity of NaNbO_3_ nanoparticles towards HER was also investigated by casting 0.24 mg/cm^2^ of sample on the electrode surface at room temperature and saturated alkaline (0.5 M KOH) electrolyte solution was used to carry out analysis. Figure 6a shows the CV measurements of NaNbO_3_ nanoparticles for HER activity, carried out in a potential window ranging from 0 to −1.4 V versus Ag/AgCl at a scan rate of 100 mV/s. The peak which is observed at ~−0.34 V could be due to the reduction of oxygen during the cathodic sweep of CV. Thereafter, hydrogen evolution reaction starts by the electrolysis of water. From Fig. 6a, it can be visualized that at 100 mV/s scan rate nanosized sodium niobate shows HER activity according to the reaction below.

$$2{{\rm{H}}}_{2}{\rm{O}}\,({\rm{l}})+2{{\rm{e}}}^{-}\to 2{{\rm{OH}}}^{-}+{{\rm{H}}}_{2}$$

Figure 6b shows the linear sweep polarization curve of the sodium niobate nanoparticles for HER. The polarization curve shows that, sodium niobate nanoparticles show onset potential equal to ~−1.0 V with respect to Ag/AgCl in alkaline medium, which is comparable to the already reported electrocatalysts^64^, but the current density obtained is low and therefore, less hydrogen is produced during the HER process. Similarly, Fig. S3a represents the LSV plot of bulk NaNbO_3_ sample. A very small current was observed as compared to nanosized NaNbO_3_, which demonstrates that small amount of hydrogen was evolved from the bulk catalyst surface. Table 1 shows the comparison of electrocatalytic OER and HER activity of NaNbO_3_ nanoparticles and bulk sample. We have used Nernst equation to convert the potential of Ag/AgCl electrode to the potential of RHE using the i.e.

$${\rm{E}}({\rm{RHE}})={\rm{E}}({\rm{Ag}}/{\rm{AgCl}})+{{\rm{E}}}^{0}({\rm{Ag}}/{\rm{AgCl}})+0.059\,{\rm{pH}}$$

**Table 1:** Comparison of electrocatalytic activity of nanosized and bulk NaNbO_3_ electrocatalyst for OER and HER.

Material	Particles size	OER current density mA/cm^2^	HER current density mA/cm^2^	Tafel slope mV/decade	
				OER	HER
Nano NaNbO_3_	10–30 nm	2.7	0.7	370	113
Bulk NaNbO_3_	500–700 nm	4 × 10^−6^	3.88 × 10^−6^	409.6	347

A small difference in the current density (i.e. 0.1 mA/cm^2^) was observed from CV and LSV. We assume that this small difference could be due to the experimental handling error in the preparation of electrode. Note that we have prepared fresh electrodes for each experiment. Reaction kinetics and mechanism for electrocatalytic HER was studied using tafel equation. Figures 6c and S3b shows the tafel plot of electrocatalytic HER on nano and bulk NaNbO_3_ catalyst surface respectively and the electrocatalytic kinetics for HER was determined by curve fitting of tafel plot. Figures 6c and S3b give the tafel slope values of 113 and 347 mV/decade for electrocatalytic HER over nano and bulk electrocatalysts respectively, which predicts the possible additional water dissociation step in HER on NaNbO_3_ nanoparticles. The tafel slope obtained for HER is comparable to the standard electrocatalysts in alkaline medium and is less as compared to many non-noble metal electrocatalysts^65^.

### Photocatalytic properties

The photocatalytic activity of the synthesized NaNbO_3_ nanoparticles was estimated by carrying out the degradation of RB organic dye by employing solar radiations as light source and efficiency of photocatalytic activity of as-prepared sample was also examined. To carry out the actual degradation experiment, several control experiments were done to determine the effect of sunlight and surface adsorption of catalysts without light source. Generally, catalytic surfaces are responsible for the photo-oxidation reaction; therefore, the adsorption phenomenon of catalyst/organic dye/pollutant is an important factor for photocatalysis. The ability of NaNbO_3_ to adsorb RB dye on its surface was evaluated in absence of sunlight. The experiment revealed that no evident adsorption is taking place over the NaNbO_3_ photocatalyst surface. The adsorption profile of the synthesized nanoparticles in absence of light irradiation is shown in Fig. S4. From the adsorption study of the nanoparticles, it was observed that after 100 min very little amount of dye has been adsorbed by NaNbO_3_ nanoparticles.

**Figure 7:**
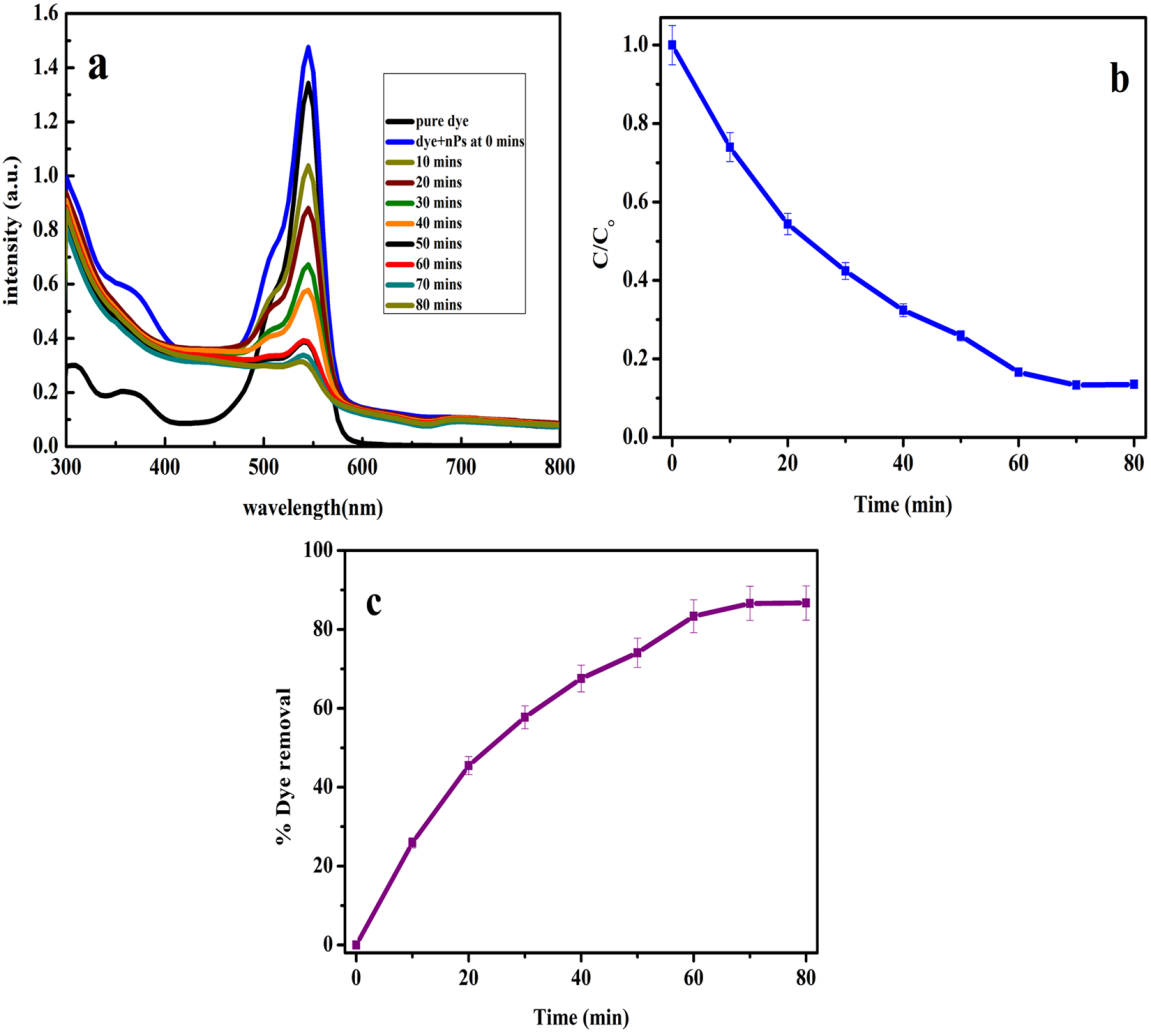
(**a**) Absorption spectra of RB dye solution during the photocatalytic reaction with NaNbO_3_, (**b**) photocatalytic efficiency of NaNbO_3_ and (**c**) percentage removal of RB dye using NaNbO_3_ nanoparticles.

Efficiency of synthesized photocatalyst was evaluated by decolourization of 1 × 10^−5^ M concentration RB dye solution for 80 min. The decolourization of RB dye was also tested in absence of the photocatalyst. From the experiments, it was determined that the RB dye does not undergo auto degradation in 80 min at pH 7.2. After 30 min in dark, the dye solution was exposed to bright sunlight and degradation was evaluated by monitoring the change in intensity of λ_max_ at 545 nm after every 10 min by using UV-vis spectrophotometer. Figure 7a shows UV-vis spectra of degradation of RB dye by NaNbO_3_ photocatalyst under sunlight. The relative intensity of λ_max_ of RB dye molecule continuously decreases with respect to time, which confirms that NaNbO_3_ photocatalysts is carrying out the degradation of RB dye. From the photocatalytic dye degradation study, it was confirmed that the synthesized sample is an active photocatalyst. Figure 7b shows the relative change in concentration (C/C_0_) of RB dye in presence of NaNbO_3_ catalyst with time. The calculations for percentage removal of dye by NaNbO_3_ photocatalysts were carried out by using equation as discussed in experimental section. Figure 7c demonstrates the RB dye removal percentage from aqueous solution and it was found that NaNbO_3_ eliminates ≈86% of dye in 80 mins when exposed to sunlight which is much higher than NaNbO_3_/ZnO heterojunction photocatalyst (75%), pristine NaNbO_3_ (55%) and TiO_2_ (60%)^65–67^.

The well-known factors which influence the ABO_3_ photocatalytic performance are structure (anionic and cationic sites), surface area, band gap, defect density and redox ability of ions present at B-site (lattice oxygen activity). In comparison to the bulk NaNbO_3_, an important reason for enhanced photocatalytic activity of NaNbO_3_ under solar irradiation in this work is due to the large surface area of NaNbO_3_ nanoparticles. The increased surface area results in production of more reaction active sites which enhances the reaction rate of the nanocatalyst.

In perovskite metal oxides, A cation sites are usually considered as catalytically inert as compared to the B-site cations and consequently, A-site cations have marginally less effect on photocatalysis as compared to the cations at site B, whose valence band and conduction band comprises of O 2p and B 3d orbitals respectively^68,69^.

It can be concluded that NaNbO_3_ based perovskite catalyst shows considerable photocatalytic activity in degradation of RB dye when irradiated with solar radiations, due the formation of NbO_6_ octahedral chains which could favour the possible delocalization of charge carriers during the catalytic reaction. To determine the active species and possible photocatalytic degradation mechanism responsible for removal of organic dye, different scavengers such as Benzoquinone (BQ), isopropanol (IP), silver nitrate (AgNO_3_) and ammonium oxalate (AO) were used. BQ, IP, AgNO_3_ and AO acts as scavengers for O_2_^•−^, OH^•^, e^−^ and h^+^ respectively. Scavengers were employed to quench different reactions carried out by different oxidising species during photocatalysis. As a result of quenching, the more photocatalytic activity is reduced by any scavenger, the more actively is the oxidising species taking part in degradation reaction. Figure S5 represents the reduction in the photocatalytic activity of the catalyst by using different scavengers. From the quenching results, it was observed that addition of BQ, IP and AgNO_3_ reduces the catalytic activity of photocatalyst to large extent. While as, addition of AO has least effect on the catalytic properties of the catalyst. The results obtained confirms the predominant role of OH^•^ in photocatalytic reaction along with photogenerated electrons and superoxide radical anion. The possible mechanism for photocatalytic degradation over the surface of NaNbO_3_ is described by following equations.

$${{\rm{NaNbO}}}_{3}+hv\to {{\rm{NaNbO}}}_{3}({{\rm{e}}}^{-}+{{\rm{h}}}^{+})$$

$${{\rm{NaNbO}}}_{3}({{\rm{h}}}^{+})+{{\rm{H}}}_{2}{\rm{O}}\to {{\rm{NaNbO}}}_{3}+{{\rm{OH}}}^{\cdot }+{{\rm{H}}}^{+}$$

$${{\rm{NaNbO}}}_{3}({{\rm{h}}}^{+})+{{\rm{OH}}}^{-}\to {{\rm{NaNbO}}}_{3}+{{\rm{OH}}}^{\cdot }$$

The conduction band electrons of NaNbO_3_ reduce the molecular oxygen to the hydroperoxyl radicals according to reaction below:

$${{\rm{NaNbO}}}_{3}+{{\rm{O}}}_{2}+{{\rm{e}}}^{-}\to {{\rm{NaNbO}}}_{3}+{{\rm{O}}}_{2}^{\cdot -}$$

$${{\rm{O}}}_{2}^{\cdot -}+{{\rm{H}}}_{2}{\rm{O}}\to {{\rm{OH}}}_{2}^{\cdot }+{{\rm{OH}}}^{-}$$

$${{\rm{OH}}}_{2}^{\cdot }+{{\rm{H}}}_{2}{\rm{O}}\to {{\rm{H}}}_{2}{{\rm{O}}}_{2}+{{\rm{OH}}}^{\cdot }$$

$${{\rm{H}}}_{2}{{\rm{O}}}_{2}\to 2{{\rm{OH}}}^{\cdot }$$

$${{\rm{OH}}}^{\cdot }+{\rm{RB}}\to {{\rm{CO}}}_{2}+{{\rm{H}}}_{2}{\rm{O}}$$

$${{\rm{NaNbO}}}_{3}+{\rm{RB}}\to {\rm{degraded}}\,{\rm{product}}$$

**Figure 8:**
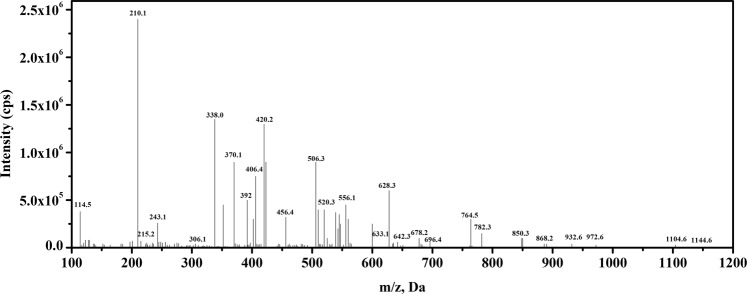
LC-MS spectrum of Rose Bengal dye after photocatalytic degradation by NaNbO_3_ nanoparticles.

**Figure 9:**
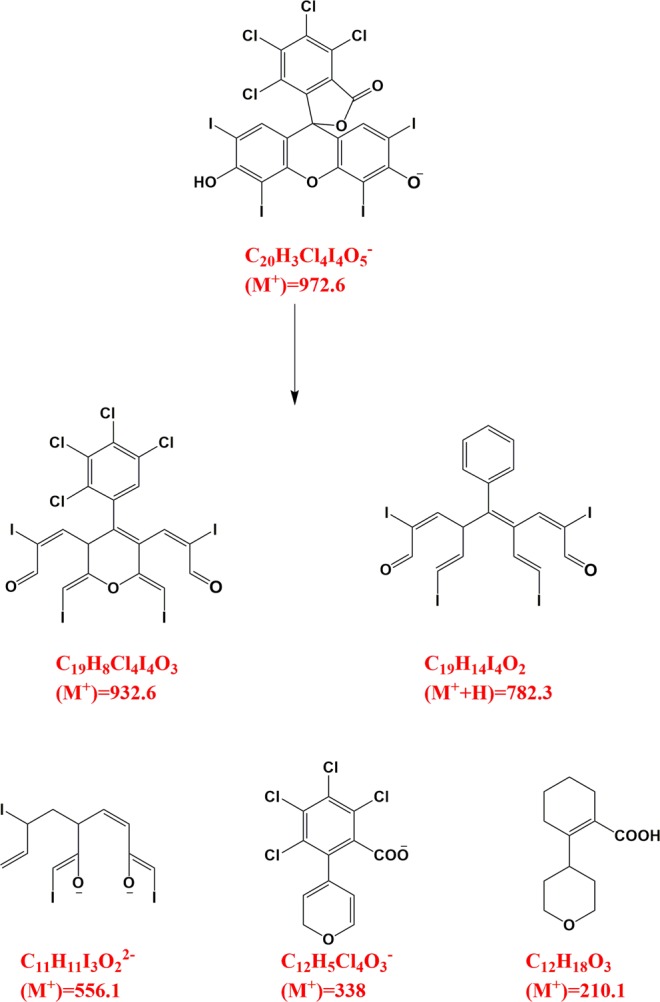
Possible degradation fragments obtained after photocatalytic degradation of Rose Bengal dye.

Under solar irradiation, the valence band electrons are excited and are transferred to its conduction band thereby, results in the creation of photoactive electron and hole pairs. The photogenerated electrons can be further used by the molecular oxygen to excite molecular oxygen for formation of activated species like superoxide^70^ thus, results in the reduced electron and hole recombination by prolonging their lifetime. The generated superoxide radical anion helps in the formation of hydroxyl radical which is an important oxidizing species taking part in the photodegradation of RB dye. The holes generated in the valance band during solar illumination may also play an active role in the degradation of RB dye. Thus, the combination of hydroxyl radical and holes vibrantly take part in photodegradation of the RB dye. Liquid chromatography mass spectrometry (LC-MS) was used to confirm the possible degradation and intermediates formed after 80 min. Sample with minimum intensity in the UV-visible spectra was used for LC-MS analysis. Figure 8 shows the mass spectra of the RB dye solution after photocatalytic reaction, which shows that after 80 min no peak corresponding to RB dye is observed confirming the degradation of the dye. The different possible fragments obtained during the process of photocatalysis are presented in Fig. 9.

**Figure 10:**
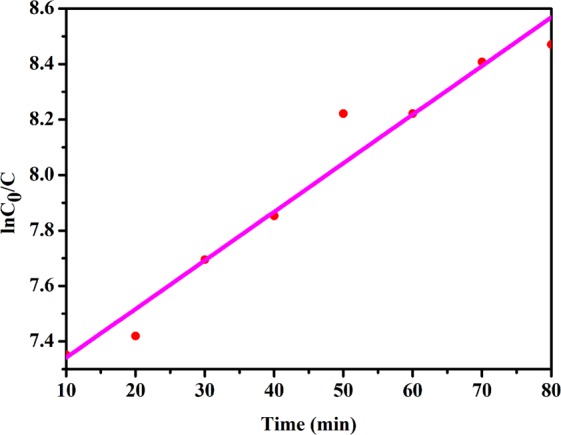
Kinetic plot of dye degradation by NaNbO_3_ nanoparticles.

The kinetics of degradation process of RB dye was modelled with the Langmuir–Hinshelwood mechanism^71^. The importance of this model is that it also covers adsorptions properties of the photocatalyst. The apparent rate constant of degradation of RB was evaluated by using Langmuir–Hinshelwood model. From experimental data, the rate constant was calculated by using linear fit and was equal to 0.018/min for NaNbO_3_ with R^2^ equal to 0.98. Equation 1 is the kinetic equation used to determine the rate constant for the photocatalytic reaction.

1 $$\mathrm{ln}\,{{\rm{C}}}_{{\rm{o}}}/{\rm{C}}=k{\rm{Kt}}=R{\rm{t}}$$

where C_o_ represents the initial concentration (molL^−1^) of dye and C corresponds to the concentration after time t and *R* represents the apparent rate constant (min^−1^) of photocatalytic dye degradation. The semi-logarithmic plot of concentration of RB dye versus time of irradiation produces a straight line as shown in Fig. 10 which indicates the degradation of the dye follows pseudo-first order reaction kinetics.

### Dielectric properties

**Figure 11:**
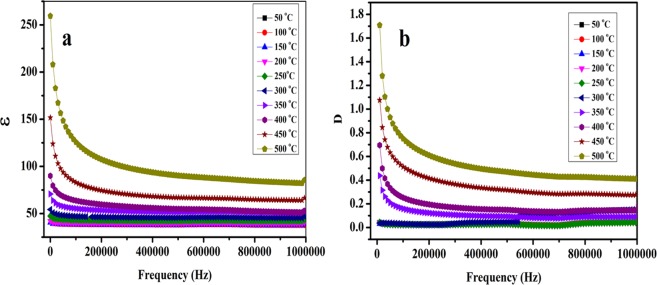
Variation of (**a**) dielectric constant and (**b**) dielectric loss of the NaNbO_3_ nanoparticles sintered at 500 °C with frequency at different temperatures.

**Figure 12:**
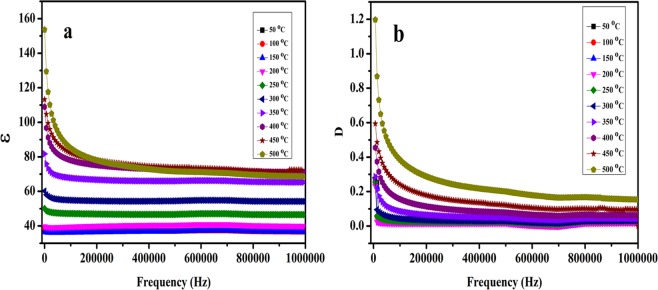
Variation of (**a**) dielectric constant and (**b**) dielectric loss of the NaNbO_3_ nanoparticles sintered at 700 °C with frequency at different temperatures.

Frequency and temperature dependent dielectric properties of NaNbO_3_ pellets sintered at two different temperatures (500 °C and 700 °C) were measured from 20 Hz to 1 MHz at different temperatures ranging from 25 °C to 500 °C. Figure S6 shows the variation of dielectric constant (ɛ) and dielectric loss (D) of the NaNbO_3_ samples sintered at 500 °C and 700 °C with frequency at room temperature. The variation of (ɛ) and (D) of NaNbO_3_ nanoparticles sintered at 500 °C and 700 °C with frequency at different temperatures (50 °C to 500 °C) is shown in Figs 11 and 12, respectively. The dielectric constant (ɛ) is found almost independent on frequency at low temperature <250 °C, however, with increase in frequency there is an appreciable decrease in dielectric loss (D) values. With increase in temperature, the change in (ɛ) and (D) with respect to frequency becomes more prominent as shown in Figs 11 and 12. Both (ɛ) and (D) have high value at low frequencies which shows decreasing trend with increase in frequency. The decrease in (ɛ) and (D) is gradual at low frequencies and becomes almost constant at higher frequencies. This declined behaviour can be explained by long-time polarization effect caused by certain structures such as space charges, dipoles which do not have ability to contribute to the overall polarization at higher frequencies^72^. At low frequencies, space charge polarization does undergo the relaxation process which follows the frequency of the applied field easily whereas, at high frequencies, these space charges do not get enough time to orient themselves along the frequencies of applied field^73^. The reason for the decrease of (ɛ) and (D) with frequency at different temperatures is that electric dipoles are not capable to follow the applied electric field as explained by Maxwell-Wagner interfacial polarization^74^. The change of (D) with respect to frequencies can be explained by Koop’s phenomenological theory^75^. Low loss value can be attributed to the nano dimensional particles^76^.

**Figure 13:**
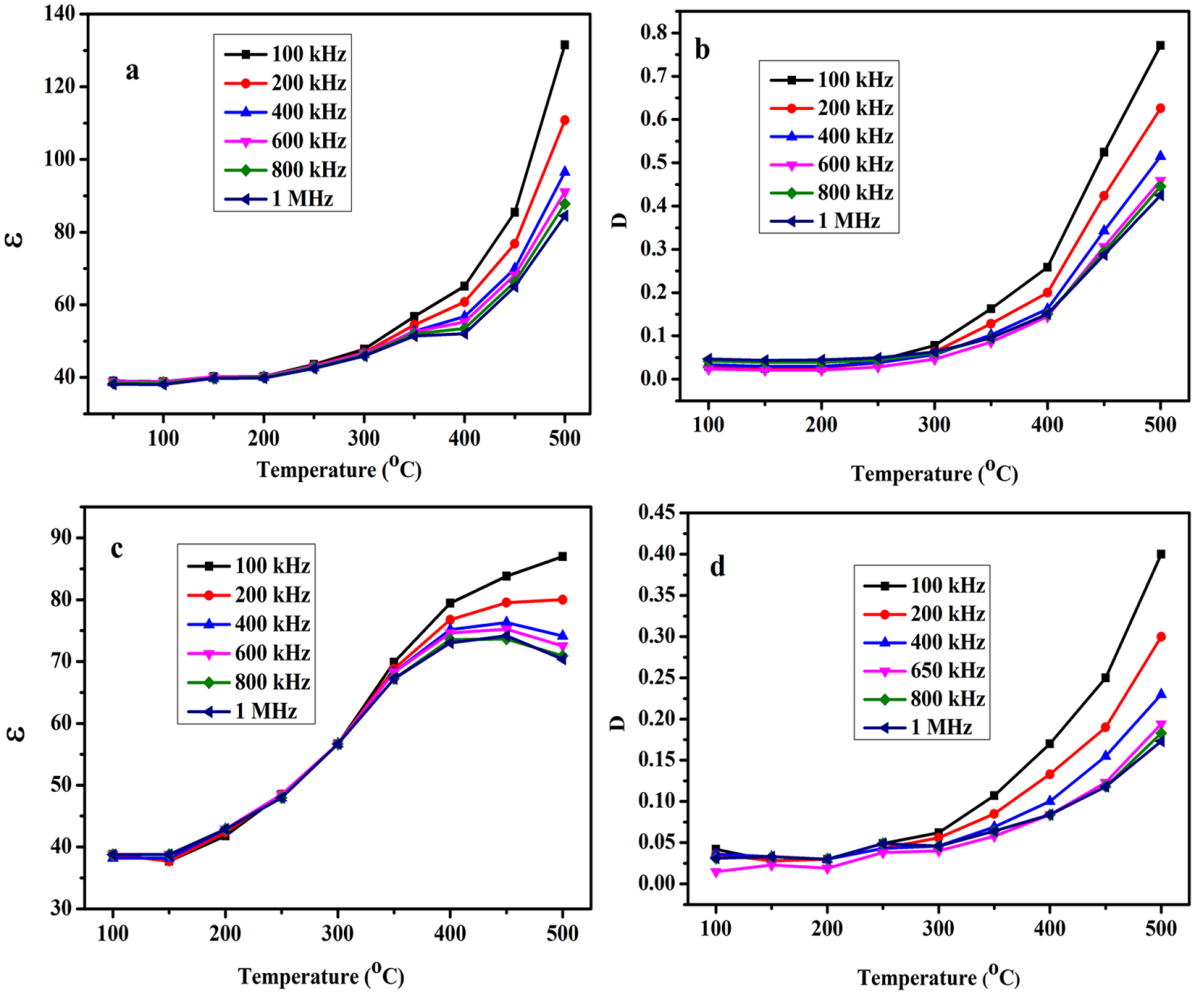
Variation of (**a**) dielectric constant, (**b**) dielectric loss of sample sintered at 500 °C (**c**) dielectric constant and (**d**) dielectric loss of sample sintered at 700 °C with temperature at different frequencies.

**Figure 14:**
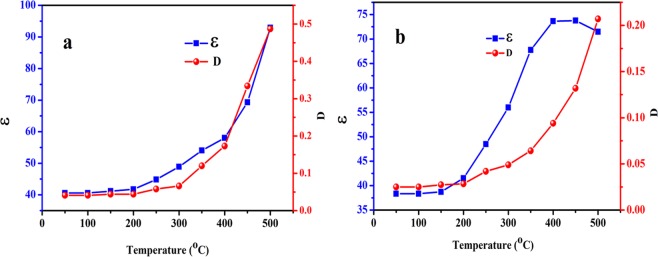
Variation of dielectric constant and dielectric loss with temperature at 500 kHz frequency of NaNbO_3_ nanoparticles sintered at (**a**) 500 °C and (**b**) 700 °C.

The variation of (ɛ) and (D) with respect to temperature measured at different frequencies (1kHz–1MHz) is shown in Fig. 13a–d for samples sintered at 500 °C and 700 °C respectively. From the results obtained, it is observed that with increase in temperature both (ɛ) and (D) increases at different frequencies. At temperature above 300 °C, both dielectric loss as well as dielectric constant increases sharply which may be the result of increased conductivity. Figure 14a,b represents the variation of (ɛ) and (D) of samples sintered at 500 °C and 700 °C with temperature at 500 kHz frequency respectively. The results obtained show that sample sintered at 500 °C show stable value of (ɛ) and low (D) upto 250 °C. Above 250 °C, both (ɛ) and (D) increases sharply, therefore, upto 250 °C NaNbO_3_ nanoparticles sintered at 500 °C could be used as stable dielectric material in electronic devices. However, the sample sintered at 700 °C shows the same effect in the properties upto 200 °C.

The dielectric constant of the NaNbO_3_ nanoparticles was found to be 41 and 38.5 for the samples sintered at 500 °C and 700 °C, respectively at 500 kHz. The increase in sintering temperature increases the crystallinity and hence the crystallite size of nanoparticles is increased. Therefore, the sample sintered at high temperature has fewer lattice defects compared to the sample having small crystallite size and low sintering temperature^77^. According to conductivity model, the ac conductivity of the materials is directly proportional to the amount of defect sites present in the material^77^. Therefore, at high sintering temperature the sample has minor defect sites and may have low conductivity values as discussed later in ac conductivity section. Dielectric constant and square root of conductivity of the materials are directly proportional to each other. From ac conductivity results, with increase in sintering temperature, the ac conductivity decreases therefore, dielectric constant of NaNbO_3_ nanoparticles decreases. Figure S7 shows the TEM images of the NaNbO_3_ nanoparticles sintered at two different temperatures. Figure S7a represents the TEM image of the sample sintered at 500 °C while as Fig. S7b shows the TEM micrograph of the sample sintered at 700 °C. The change in (ɛ) and (D) at different frequencies and temperatures are tabulated in Supplementary Tables 2S–5S. This change in (ɛ) and (D) with temperature of the samples sintered at different temperatures is plotted in Supplementary Fig. S8 which shows that with increase in temperature, the difference in the (ɛ) and (D) at initial (20 Hz) and final (1 MHz) frequency increases.

### AC conductivity

**Figure 15:**
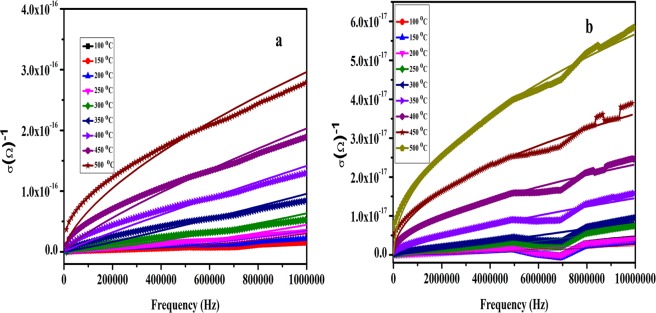
Variation of conductivity with frequency at different temperatures of NaNbO_3_ nanoparticles sintered at (**a**) 500 °C and (**b**) 700 °C.

Figure 15a,b shows the dependence of ac conductivity on frequency of the samples sintered at 500 °C and 700 °C respectively. For the sample (sintered at 500 °C), upto temperature <250 °C there is negligible change in conductivity of the NaNbO_3_ nanoparticles. However, above 250 °C temperature, the conductivity increases sharply throughout the frequency range. The increase in conductivity is ascribed to the increase in the number of charge carriers due to formation of large oxygen vacancies in the lattice at high temperatures^78^. Similarly, for the sample (sintered at 700 °C), the conductivity almost remains independent on frequency upto 300 °C, however, above this temperature, the conductivity shows sharp increment with increase in frequency. Fitting of power law (σ = Aω^η^) was used to understand the variation of conductivity of the samples with frequency at different temperatures. The value of “η” determines the mechanism of the conductivity. For η > 1, conductivity follows the Maxwell Wagner (M-W) relaxation process^79^, while as, for η < 1 conduction process follows correlated barrier hopping (CBH) mechanism^79^. By applying power law to the conductivity both the mechanisms were followed by NaNbO_3_ nanoparticles at different temperatures. The NaNbO_3_ nanoparticles sintered at different temperatures show η > 1 upto 350 °C, suggesting that samples follow M-W mechanism of conduction. However above 350 °C, η < 1 therefore CBH mechanism is responsible for the conduction. The conduction mechanism is clearly visible in the frequency range 20Hz–1MHz, where the η starts decreasing with increase in temperature suggesting that CBH process starts to dominate at high temperature and above 350 °C, CBH completely overcomes the M-W mechanism. This may be since at higher temperature the charge carriers get enough thermal energy to show the conduction barrier hopping process. The value of η at different temperatures is tabulated in Table 6S.

## Conclusion

In summary, high surface area nanosized sodium niobate has been successfully fabricated at low temperature by facile polymeric citrate precursor route. The synthesized nanoparticles were characterized by using XRD, TEM, XPS and BET surface area analysis. The synthesized NaNbO_3_ nanoparticles were directly employed as electrode material for OER and HER activity, which shows promising results like significant current densities, comparable onset potential and tafel slopes for OER and HER with respect to state of art electrocatalysts. Electrocatalytic properties of nanosized and bulk NaNbO_3_ samples were carried out which demonstrated that nanosized NaNbO_3_ show better activity as compared to bulk counterpart. Photocatalytic studies were carried out for over 80 min that showed enhanced catalytic degradation (~86%) of the organic dye (RB) on the surface of NaNbO_3_. The substantial electrocatalytic and photocatalytic performances were ascribed to the large surface area of NaNbO_3_ catalyst. The change of dielectric properties with operating frequency, temperature and sintering temperature have been discussed considering Maxwell-Wagner and Koop’s theory. It was observed that with increase in sintering temperature from 500 °C to 700 °C there is slight reduction of dielectric constant from 41 to 38.5 at 500 kHz frequency. Conductivity process of the samples was understood by applying power law fitting. The present study offers strategy for the design of new multifunctional materials for energy conversion techniques.

## Material and Methods

### Materials

Citric acid (Merck), Ethylene glycol (Merck), Niobium chloride (Alfa Aesar), Sodium hydroxide (Merck), Rose Bengal (Thomas Baker), Benzoquinone (BQ) (Merck), AgNO_3_ (Merck), Ammonium oxalate (AO) (Merck) and Isopropanol (Merck) were of analytical grade and were used without further purification.

### Synthesis of Nano-sized NaNbO_3_

NaNbO_3_ nanoparticles were synthesized at low temperature by using polymeric citrate precursor method. 0.1 M aqueous solution of sodium hydroxide was prepared in deionised water. Citric acid was added in 25 ml of aqueous sodium hydroxide solution and was stirred for about 10 min. To this solution, 0.025 moles of niobium chloride was added and stirred for 3 h followed by addition of ethylene glycol. Then, the solution mixture was stirred and heated at 55 ± 5 °C continuously until the formation of viscous gel. Then, the reaction mixture was placed inside the muffle furnace and temperature was increased up to 135 °C for 20 h. After 20 hrs, temperature was further raised and kept at 300 °C for 2 hrs. A black mass referred to as precursor was obtained. The as-obtained precursor was grinded and calcined in air at 500 °C for 12 h and was used for further characterization and studies. The molar ratio of Citric acid: metal ion: Ethylene glycol during the reaction was 40: 1: 10.

### Synthesis of bulk NaNbO_3_

In a typical synthesis process, 10 N NaOH solution was prepared and was kept on stirring. To this solution 0.025 moles of Nb_2_O_5_ was added and stirred for 1 h. The reaction mixture was then shifted to 50 ml teflon lined autoclave and was heated in muffle furnace at 150 °C for 4 h. The reaction mixture was then left to cool down naturally. Final product was collected by centrifugation and washing.

### Characterization

The powder X-ray diffraction technique (PXRD) was used to determine the crystallinity and phase purity. Rikagu X-ray diffractometer equipped with Cu Kα radiation (λ = 1.5406 Å) was used to obtain the diffraction patterns at the normal scans with a step size of 0.5 °/s and a step time of 1 s with 2θ ranging from 20° to 70°. The XRD pattern and peak positions obtained were compared with the JCPDS standard files to identify the phase and lattice parameters of the synthesized material. To estimate the exact shape and size of the as-synthesized nanoparticles, Transmission electron microscopic (TEM) study was carried out by using FEI Technai G^2^ 20 HRTEM with an accelerating voltage of 200 kV. To exactly determine the chemical composition and binding energies of different components of the synthesized material, the X-ray photoelectron spectroscopic studies were carried out by using EAC200 SPHERA 547 having Mg Kα as radiation source. BET surface area analyser (Nova 2000e, Quantachrome Instruments Limited, USA) was employed to estimate the specific surface area and the pore size of the sample in the presence of liquid nitrogen (77 K). To remove the adsorbed gases and moisture from the sample, vacuum degassing was carried out for 3 h at 200 °C. Specific surface area was determined by using Multipoint BET equation and pore size distribution was determined by using Dubinin-Astakhov (DA) method. All the calculations made in the BET study are done automatically by the instrument using the software-based equations to compute specific surface area and pore size distributions.

### Electrocatalytic measurements

The electrochemical measurements were carried out with CHI 660E, China electrochemical analyser having a standard three electrode cell testing system at 30 °C. The synthesized sodium niobate nanoparticles were employed as working electrode while, Ag/AgCl and Pt wire were used as reference and counter electrode respectively. The working electrodes were prepared by putting a drop of sample slurry over the surface of glassy carbon electrode (GCE) and then dried prior to use it. The slurry was prepared by using 5.0 mg NaNbO_3_ electro-catalyst with 0.025 ml Nafion and 1 ml propanol. The resulting mixture was then sonicated for 30 minutes to get the homogeneous mixture. The loaded mass of the NaNbO_3_ electro-catalysts on the GCE was ~0.2 mg/cm^2^. The surface area of electrode was 0.07 cm^2^, which was used to calculate the current density of electrode materials. Cyclic voltammetry (CV) was used to determine hydrogen evolution reaction and oxygen evolution reaction activity of NaNbO_3_ electrode at scan rate 100 mV/s in 0.5 M KOH electrolyte. CV measurements of sodium niobate nanoparticles for HER and OER were evaluated in a peak window range from 0 to −1.4 V. Linear sweep voltammetry curve (LSV) was obtained in cathodic as well as anodic directions and was used to calculate the onset potential and current density for HER and OER. The Nernst equation can be employed for the conversion of the potential of OER and HER versus Ag/AgCl electrode to RHE at room temperature i.e.

$$({E}_{({\rm{RHE}})}={E}_{({\rm{Ag}}/{\rm{AgCl}})}+0.059\,{\rm{pH}}+0.197\,{\rm{V}}).$$

### Photocatalytic experiment

The activity of NaNbO_3_ nanoparticles as photocatalyst was assessed by monitoring the degradation of (RB) as a probe reaction. Photocatalytic reaction was carried out in presence of sunlight at an atmospheric temperature (35 °C). 20 mg nanoparticles were dispersed in 50 ml RB dye solution having 1 × 10^−5^ M concentration. The suspension obtained was stirred continuously in the dark conditions for 30 min to attain the adsorption desorption equilibrium between organic dye and the catalyst. After reaching adsorption desorption equilibrium, photocatalytic reaction was initiated by irradiation with sunlight. The spectrum was recorded at the interval of 10 min during the total period of 80 mins for analysis. Other experiments were also carried out either in absence of catalysts or under dark conditions to authorize that the degradation reaction is carried out by photocatalysis only. The process of photodegradation of RB dye was observed by measuring the characteristic change in absorption intensity at 545 nm using UV-visible spectrophotometer. The percentage photocatalytic degradation was calculated using following equation.

2 $${\bf{D}}({\boldsymbol{ \% }})=({{\bf{C}}}_{{\bf{0}}}-{\bf{C}}/{{\bf{C}}}_{{\bf{0}}})\times {\bf{100}}$$

where C_0_ is the RB dye concentration at t = 0 i,e., after attainment of adsorption-desorption equilibrium before irradiation and C is the concentration of dye after time interval t. Liquid chromatography mass spectroscopy (LC-MS) of the dye solution was carried out to attest whether the dye has been degraded or not.

### Dielectric measurements

Dielectric measurements of the NaNbO_3_ samples were carried out in air at temperatures ranging from 25–500 °C over a frequency range of 20Hz–1MHz. HF-LCR meter (6505 P, Wayne Kerr Electronics, UK) was used to carry out the dielectric measurements. For dielectric measurements, principle of parallel plate capacitor was used. These measurements were carried out by using 8 mm disk shaped pellet with a thickness of 0.5 mm prepared by applying uniaxial pressure of 5 tons and using 5% polyvinyl alcohol (PVA) as a binder. The pressure was applied using the KBr press Model M-5 (technosearch instruments). The prepared pellets were sintered at 500 °C and 700 °C temperatures to remove the binder from the sample. The pellets were coated with thin layer of silver paint (Ted Pella, Inc.) to form conducting contacts which act as electrode. Virtual instrument package LABVIEW (National Instruments) in interference with LCR meter was used to collect the data.

## Supplementary information

Supplementary Material
