# Supplementary material for: Electrocatalytic and Enhanced Photocatalytic Applications of Sodium Niobate Nanoparticles Developed by Citrate Precursor Route
Source: Sci Rep. 2019 Mar 14;9:4488. doi: 10.1038/s41598-019-40745-w (PMC6418189; doi:10.1038/s41598-019-40745-w)
Supplement: Supplementary file 1 — Supplementary Material [file 41598_2019_40745_MOESM1_ESM.docx]

**Supplementary Material**

**Electrocatalytic and Enhanced Photocatalytic Applications of Sodium Niobate Nanoparticles Developed by Citrate Precursor Route**

**Umar Farooq^1^,** **Ruby Phul^1^, Saad M. Alshehri^2^, Jahangeer Ahmed^2^ and Tokeer Ahmad^1^***

^1^Nanochemistry Laboratory, Department of Chemistry, Jamia Millia Islamia, New Delhi-110025, INDIA

^2^Department of Chemistry, College of Science, King Saud University, Riyadh 11451, Saudi Arabia

**Mechanism for the synthesis of NaNbO_3_ by polymeric citrate precursor route.**

The detailed mechanism of synthesis of NaNbO_3_ by polymeric citrate precursor route is given somewhere else in ref^37^. In this method metal oxide nanoparticles are synthesized by using a multifunctional organic acid like citric acid which acts as chelating agent. The chelating agent along with the diol like ethylene glycol forms a stable complex with metal ion. The soluble complex is formed which on evaporation of the solvent changes into a highly viscous gel. With further heating process the gel is turned into black mass known as precursor. Finally, this precursor is heated at 500 ^o^C to get NaNbO_3_ as final product.





**Figure S1:** XRD pattern of black precursor obtained at 300 ^o^C.


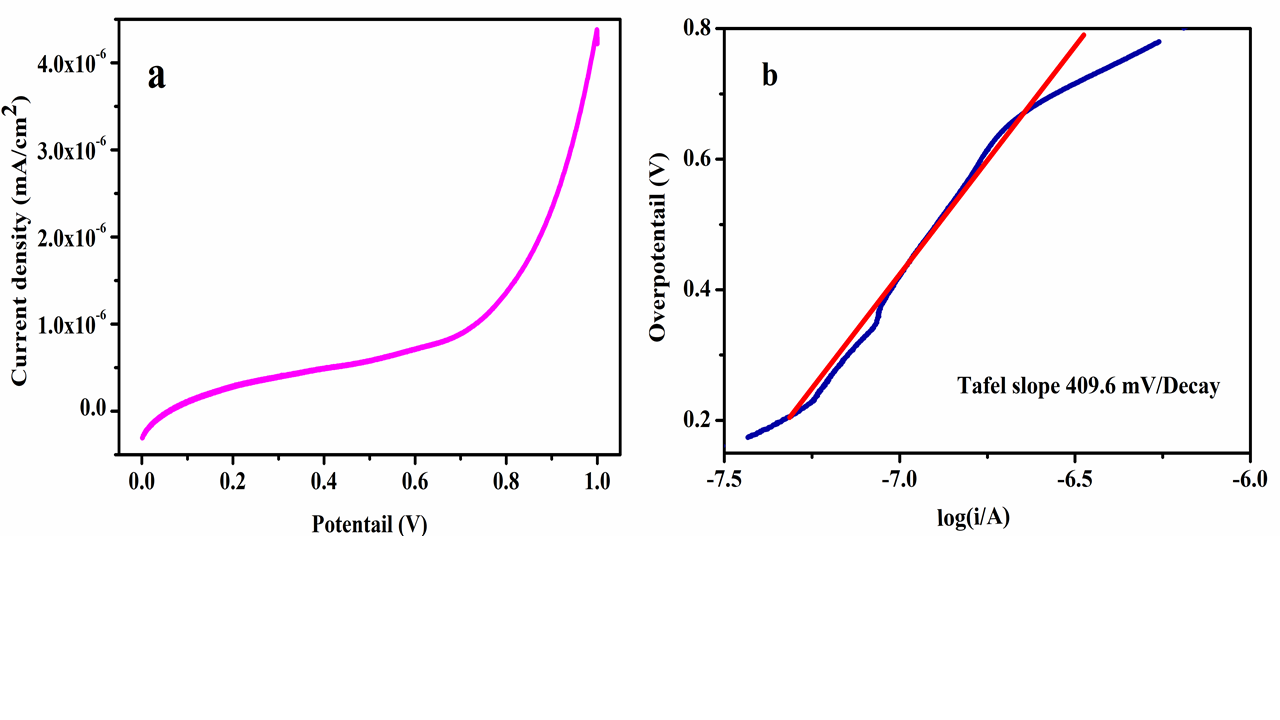


**Figure S2.** (a) LSV curve in O_2_ saturated 0.1 M KOH solution at a scan rate of 100 mVs^-1^ and (b) Tafel plot of bulk NaNbO_3_ for OER activity.


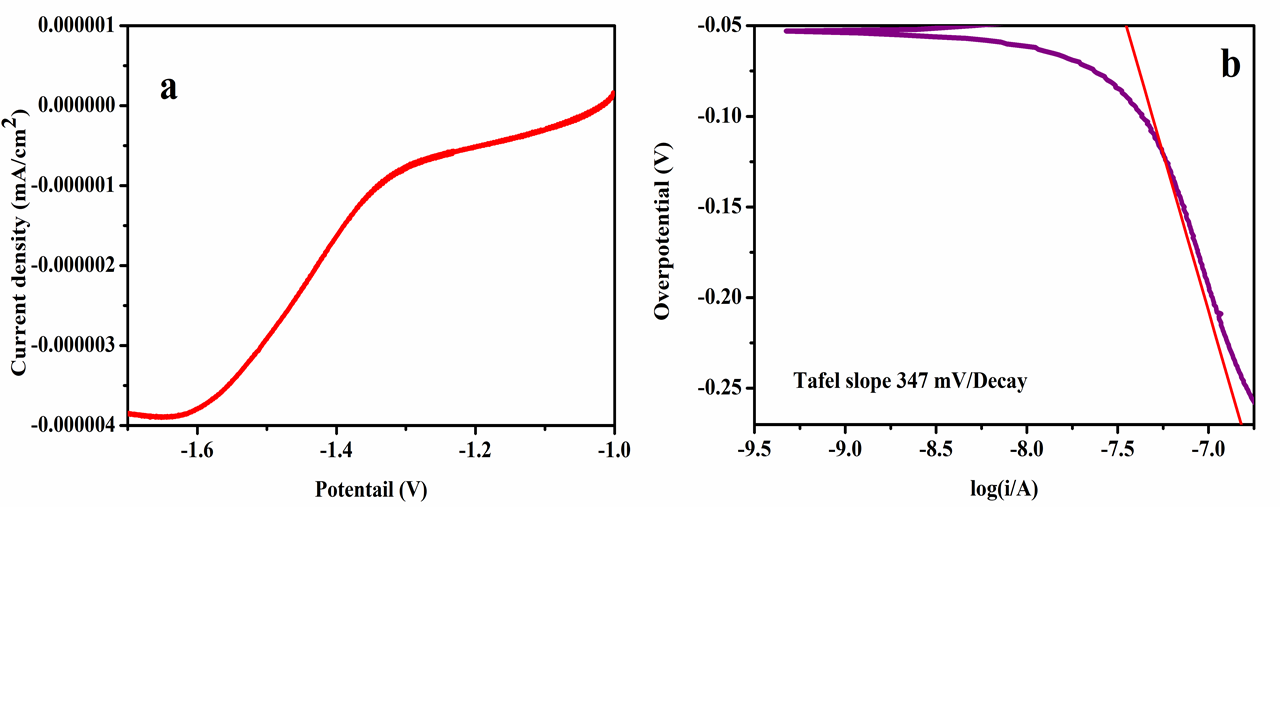


**Figure S3.** (a) LSV curve in O_2_ saturated 0.1 M KOH solution at a scan rate of 100 mVs^-1^ and (b) Tafel plot of bulk NaNbO_3_ for HER activity.





**Figure S4:** Adsorption profile of the NaNbO_3_ nanoparticles in dark.





**Figure S5**. Effects of scavengers on the photodegradation efficiency of RB dye using NaNbO_3_ Photocatalyst.


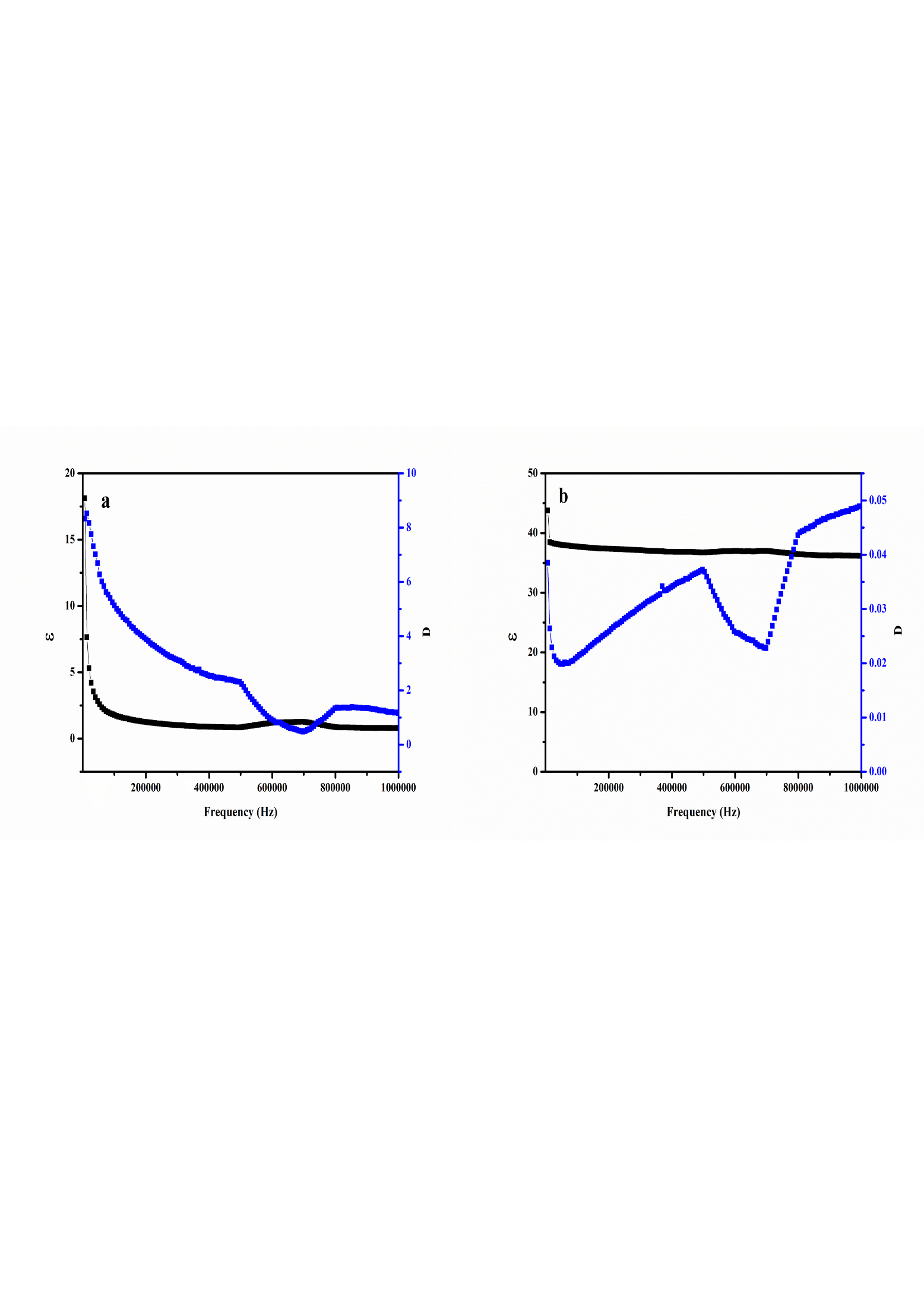


**Figure S6:** Room temperature variation of dielectric constant and dielectric loss of the samples sintered at (a) 500 °C and (b) 700 °C with frequency.

**Table 2S**: Shows the difference of the dielectric constant at different temperatures for the samples sintered at 500°C.

|  |  | |  |
| --- | --- | --- | --- |
| Temperature/^o^C | Frequency | | Change |
|  | **20Hz** | **1MHz** | **Δɛ** |
| 100 | 41.32 | 38.13 | 3.19 |
| 150 | 40.95 | 38.13 | 2.82 |
| 200 | 42.33 | 39.96 | 2.37 |
| 250 | 47.94 | 42.5 | 5.44 |
| 300 | 54.91 | 45.92 | 8.99 |
| 350 | 71.17 | 51.42 | 19.75 |
| 400 | 90.93 | 52.04 | 38.89 |
| 450 | 152.49 | 65.58 | 86.91 |
| 500 | 261.78 | 84.59 | 177.19 |

**Table 3S**: Shows the difference of dielectric loss at different temperatures for the samples sintered at 500°C.

| Temperature/^o^C | Frequency | | Change |
| --- | --- | --- | --- |
|  | **20Hz** | **1MHz** | **ΔD** |
| 100 | 0.043 | 0.047 | -0.004 |
| 150 | 0.034 | 0.044 | -0.01 |
| 200 | 0.046 | 0.045 | 0.001 |
| 250 | 0.09 | 0.049 | 0.041 |
| 300 | 0.187 | 0.062 | 0.125 |
| 350 | 0.44 | 0.09 | 0.35 |
| 400 | 0.7 | 0.15 | 0.55 |
| 450 | 1.086 | 0.28 | 0.806 |
| 500 | 1.72 | 0.42 | 1.3 |

**Table 4S:** Shows the difference of dielectric constant at different temperatures for the samples sintered at 700°C.

| Temperature/^o^C | Frequency | | Change |
| --- | --- | --- | --- |
|  | **20Hz** | **1MHz** | **Δɛ** |
| 100 | 39.61 | 34.71 | 4.9 |
| 150 | 36.88 | 37.03 | -0.15 |
| 200 | 39.92 | 40.36 | -0.44 |
| 250 | 50.58 | 47.07 | 3.51 |
| 300 | 60.69 | 54.82 | 5.87 |
| 350 | 82.07 | 66.21 | 15.86 |
| 400 | 109 | 72.28 | 36.72 |
| 450 | 114.38 | 73.68 | 40.7 |
| 500 | 155.12 | 70.52 | 84.6 |

**Table 5S:** Shows the difference of dielectric loss at different temperatures for the samples sintered at 700°C.

| Temperature/^o^C | Frequency | | Change |
| --- | --- | --- | --- |
|  | **20Hz** | **1MHz** | **ΔD** |
| 100 | 100 | 0.024 | 0.02 |
| 150 | 150 | 0.05 | 0.02 |
| 200 | 200 | 0.02 | 0.01 |
| 250 | 250 | 0.23 | 0.02 |
| 300 | 300 | 0.25 | 0.03 |
| 350 | 350 | 0.28 | 0.03 |
| 400 | 400 | 0.28 | 0.046 |
| 450 | 450 | 0.45 | 0.068 |
| 500 | 500 | 0.6 | 0.102 |


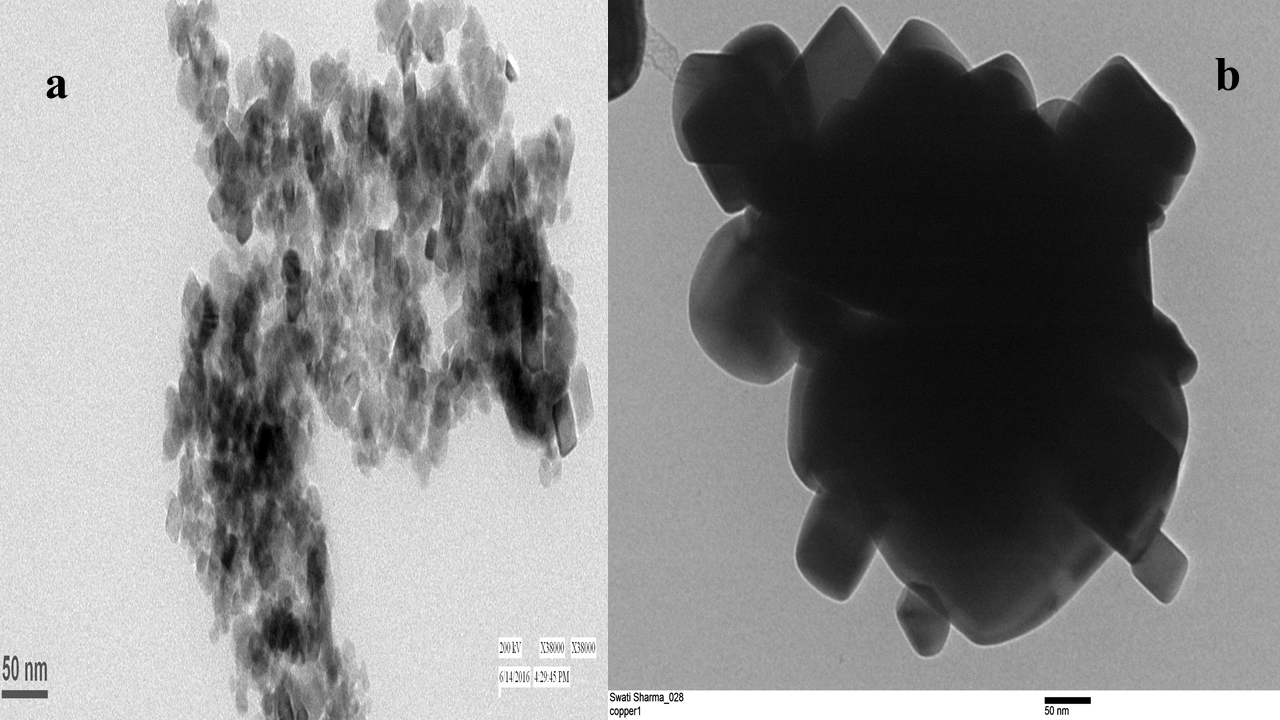


**Figure S7**: TEM micrograph showing effect of sintering temperature at (a) 500 ^o^C and (b) 700 ^o^C on NaNbO_3_ nanoparticles.


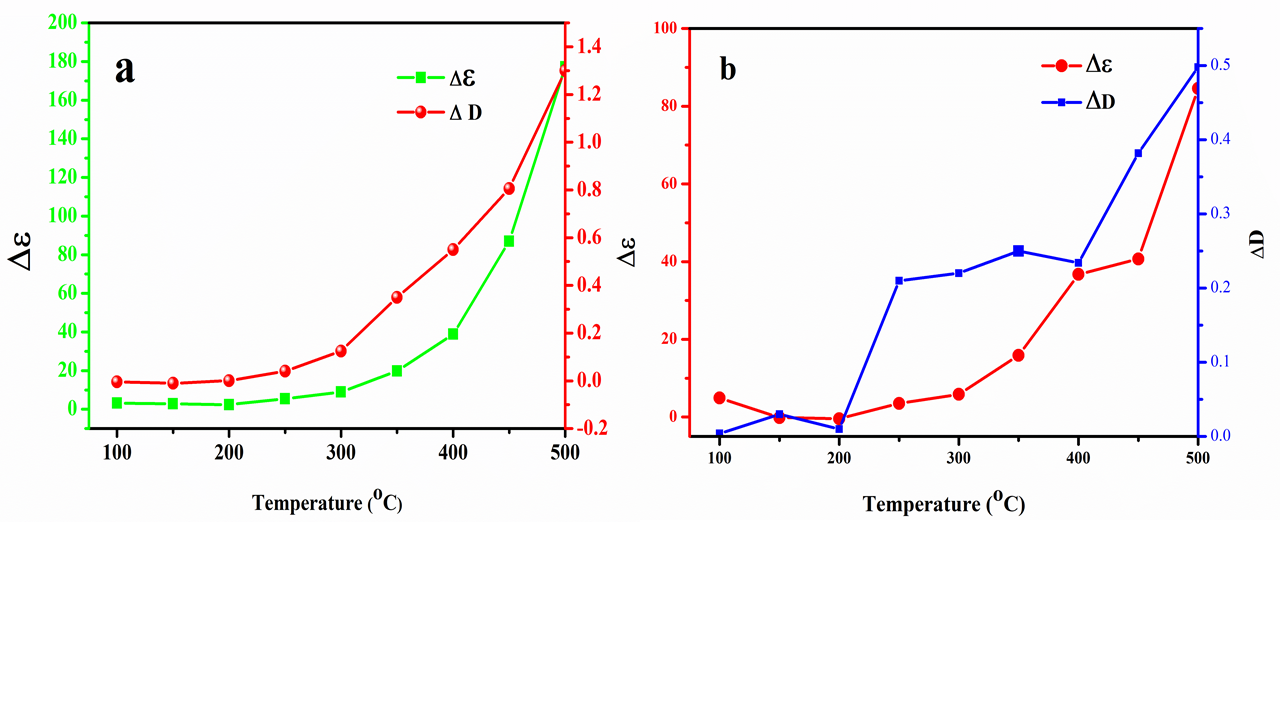


**Figure S8:** Variation in the dielectric constant and dielectric loss between 2Hz and 1MHz at different temperatures.

**Table 6S:** Shows the value of η_500_and η_700_ for the sample sintered at 500 °C and 700°C

| Temperature (°C) | η_500_ | η_700_ |
| --- | --- | --- |
| 100 | 1.95454 | 1.01996 |
| 150 | 1.90059 | 1.55926 |
| 200 | 1.74691 | 1.47286 |
| 250 | 1.61467 | 1.45926 |
| 300 | 1.35446 | 1.04914 |
| 350 | 1.10227 | 1.00926 |
| 400 | 0.91901 | 0.56932 |
| 450 | 0.80076 | 0.55926 |
| 500 | 0.6461 | 0.50935 |
